# Association Of Plasma And Urinary Mutant DNA With Clinical Outcomes In Muscle Invasive Bladder Cancer

**DOI:** 10.1038/s41598-017-05623-3

**Published:** 2017-07-17

**Authors:** K. M. Patel, K. E. van der Vos, C. G. Smith, F. Mouliere, D. Tsui, J. Morris, D. Chandrananda, F. Marass, D. van den Broek, D. E. Neal, V. J. Gnanapragasam, T. Forshew, B. W. van Rhijn, C. E. Massie, N. Rosenfeld, M. S. van der Heijden

**Affiliations:** 1 Cancer Research UK Cambridge Institute, University of Cambridge, Li Ka Shing Centre, Robinson Way, Cambridge, CB2 0RE UK; 2 Cancer Research UK Cambridge Cancer Centre, University of Cambridge, Li Ka Shing Centre, Robinson Way, Cambridge, CB2 0RE UK; 3 Academic Urology Group, Department of Surgery & Oncology, University of Cambridge, Box 279 (S4), Cambridge Biomedical Campus, Cambridge, CB2 0QQ UK; 4 Division of Molecular Carcinogenesis, Netherlands Cancer Institute, Plesmanlaan 121, Amsterdam, 1066 CX The Netherlands; 5 Department of Clinical Chemistry, Netherlands Cancer Institute, Plesmanlaan 121, Amsterdam, 1066 CX The Netherlands; 6 Nuffield Department of Surgery, Old Road Campus Research Building, University of Oxford, Oxford, OX3 7DQ UK; 7 UCL cancer Institute, Huntley St, Camden Town, London, WC1E 6DD UK; 8 Department of Surgical Oncology (Urology), Netherlands Cancer Institute, Plesmanlaan 121, Amsterdam, 1066 CX The Netherlands; 9 Department of Medical Oncology, Netherlands Cancer Institute, Plesmanlaan 121, Amsterdam, 1066 CX The Netherlands

## Abstract

Muscle Invasive Bladder Cancer (MIBC) has a poor prognosis. Whilst patients can achieve a 6% improvement in overall survival with Neo-Adjuvant Chemotherapy (NAC), many do not respond. Body fluid mutant DNA (mutDNA) may allow non-invasive identification of treatment failure. We collected 248 liquid biopsy samples including plasma, cell pellet (UCP) and supernatant (USN) from spun urine, from 17 patients undergoing NAC. We assessed single nucleotide variants and copy number alterations in mutDNA using Tagged-Amplicon- and shallow Whole Genome- Sequencing. MutDNA was detected in 35.3%, 47.1% and 52.9% of pre-NAC plasma, UCP and USN samples respectively, and urine samples contained higher levels of mutDNA (p = <0.001). Longitudinal mutDNA demonstrated tumour evolution under the selective pressure of NAC e.g. in one case, urine analysis tracked two distinct clones with contrasting treatment sensitivity. Of note, persistence of mutDNA detection during NAC predicted disease recurrence (p = 0.003), emphasising its potential as an early biomarker for chemotherapy response.

## Introduction

Bladder Cancer (BC) is the most common malignancy of the urinary tract^1^. Approximately 25% of BC patients have Muscle Invasive Bladder Cancer (MIBC) at diagnosis^2^. The presence of MIBC signifies aggressive disease with a significant risk of metastatic progression. Even after definitive treatment (e.g. radical cystectomy – surgical removal of the bladder, adjacent organs and their lymphatic drainage) overall survival (OS) is on average 50% over 5 yrs^3^. Meta-analyses show that cisplatin-based neo-adjuvant chemotherapy (NAC) can improve 5-year absolute OS by 6%^4–6^.

Despite maximum treatment with NAC and radical cystectomy, many patients do not respond to NAC^7^ and MIBC patients often recur within 2 years^8^. Once disease has progressed outside the bladder, OS survival is traditionally poor. However, a number of new agents, including immunotherapy and targeted therapies, are being tested in large clinical trials. There are currently no verified predictive biomarkers of response to cisplatin based NAC in MIBC that could be utilised prospectively, though some studies have shown initial promise using gene expression^9^ or gene mutation status^10^, DNA repair status^11^ or detecting proteins identified by gene expression analysis^12^. Furthermore, biomarkers to predict outcome during NAC are lacking and the predictive capacity of cystoscopy or radiological examination is limited^13^. In patients not responding to NAC, definitive treatment could be expedited or other systemic treatments investigated in the neo-adjuvant setting. Of note, the recent success of immune checkpoint inhibitors^14^ provides a possible alternative non-cross-resistant systemic treatment for these patients and trials with checkpoint inhibitors have recently been initiated in the peri-operative setting.

Circulating tumour DNA (ctDNA) offers a minimally-invasive means to monitor tumour status. The half-life of circulating cell-free DNA is reportedly less than 2 hours^15–17^ and the allele fraction ratio of mutant:wild type DNA (AF) has been shown to reflect tumour burden^17–19^. The translational potential for mutant DNA in body fluids (mutDNA, so called as we analysed both cell-free DNA and cellular DNA) could be even greater in bladder cancer due to the possibility of monitoring mutDNA in urine^20^, a peripheral fluid that truly can be collected ‘non-invasively’. In BC, the close proximity of tumour to the peripheral fluid reservoir might be expected to lead to a greater accumulation of tumour derived DNA.

Recent reports have shown that mutDNA is detectable in the plasma and the urine of patients with BC. Using the Affymetrix Oncoscan assay, Togneri *et al*. demonstrated that mutation profiles present in FFPE tumour specimens were mirrored in matched urinary samples from patients with Non-Muscle Invasive Bladder Cancer^21^. Ward *et al*. used digital PCR (dPCR) and NGS to analyse the somatic mutation status of cell pellets obtained by centrifuging urine samples (UCP) in 120 primary bladder cancer patients and 89 patients post transurethral resection (TUR), and detected mutant DNA in 70% of cases^22^. Furthermore, Birkenkamp-Demtröder *et al*. detected mutant DNA in plasma and urine of patients with BC by sequencing tumour specimens to design personalised droplet dPCR probes for use in peripheral fluids. In 4/6 patients, personalised probes detected plasma ctDNA several months before clinical progression^23^. These studies mostly focus on non-invasive BC, which are often driven by different pathways than MIBC^24^, and none compared plasma, UCP and USN sampling methods.

The application of mutDNA in BC represents an exciting opportunity for clinical impact in MIBC and, despite recent efforts, remains relatively unexplored. Here, we aimed to utilise a combination of Tagged-AMplicon Sequencing (TAm-Seq) and shallow Whole Genome Sequencing (sWGS) to interrogate the longitudinal dynamics of mutDNA found in peripheral samples from 17 patients with MIBC undergoing NAC. MutDNA presence and levels were compared between matching plasma, UCP and USN samples in order to determine the optimum sample type for mutDNA analysis. Furthermore, early mutDNA levels (i.e. from samples extracted pre-NAC and immediately before the second cycle of NAC) were correlated with response to NAC, as well as recurrence status. Finally, longitudinal analysis of all peripheral sample types was employed to track disease progression, and to identify tumour dynamics throughout NAC.

## Results

### Patient recruitment for longitudinal analysis of mutDNA kinetics

**Figure 1:**
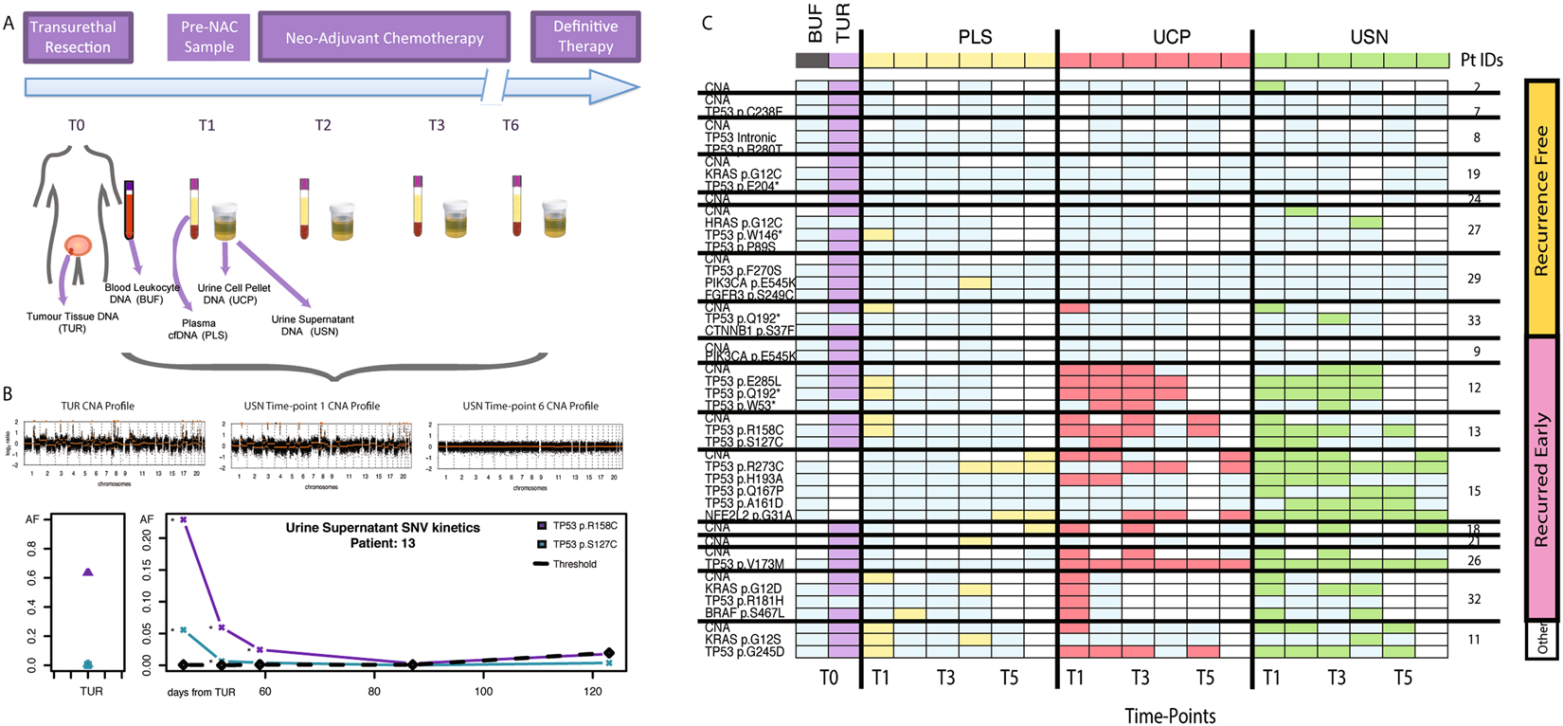
Analysis of Longitudinal mutDNA kinetics in MIBC. (**A**) Study design. 17 patients were enrolled at the NKI for mutDNA analysis whilst undergoing NAC. TUR was performed at the NKI or at regional institutions. PLS, UCP and USN were taken on one occasion before the initiation of NAC and subsequently on each chemotherapy visit, prior to definitive therapy. (**B**) Examples of longitudinal SNV and CNA analysis performed for each sample are shown. (**C**) Grid depicting mutDNA detection across all patients and time-points. The y-axis shows patients grouped by recurrence status, (right-side) and individual mutations (left-side). The x-axis shows sample-types (top) and time-points (bottom). Each cell of the grid indicates the result of a mutation analysis at that time-point (mutant-time-point analysis). White cells correspond to unavailable samples and light blue cells to samples where analysis did not detect mutDNA. Purple, yellow, red and green cells correspond to TUR, PLS, UCP and USN samples (respectively) where a mutDNA was detected. No mutations were detected in BUF (Grey). Raw AFs for the grid are provided in Supplementary Table 4.

Patients attending the Netherlands Cancer Institute (NKI) for cisplatin-based NAC were recruited between March 2014 and October 2015. We analysed 282 samples from 17 patients with MIBC, including 17 tumour tissue samples (16 FFPE TUR and 1 cystectomy; TUR tissue from patient 15 was unavailable), 17 white blood cell samples (buffy coat, BUF) and 248 body fluid samples (86 plasma, 78 UCP and 84 USN samples), spanning 86 distinct time-points in total. TUR samples were requested from referring hospitals whilst peripheral samples were collected at the NKI prior to administration of each cycle of NAC (Fig. 1A). Patients were followed up for a median of 742 days (487–952 days) following initiation of NAC and 588 days (463–851 days) following definitive therapy. Details of the patient demographics, tumour characteristics and treatment are outlined in Table 1. DNA extraction failed in 2 samples (1 plasma and 1 USN). DNA concentration was measured by a dPCR assay targeting the *RPP30* gene using a 97 bp amplicon^19^. Excluding the two failed samples, we obtained a median of 5,296 amplifiable copies/ml (ranging from 101 to 937,000 amplifiable copies/ml), with the highest extraction yields from UCP, USN, then plasma samples (respective medians in amplifiable copies/ml; 61,600, 5,870 and 3,550, Supplementary Figure 3).

**Table 1:** Demographics of 17 MIBC patients.

Pt IDs	Age at TUR	Sex	No. of Samples Collected					TUR Grade	TUR & Imaging Stage	Post TUR Diagnostic Cystoscopy	Definitive Treatment	Final Pathology	Time to Recurrence
			TUR	BUF	PLS	UCP	USN						
2	57	M	1	1	4	4	4	3	T3N0M0	3	CR	—	
7	48	F	1	1	6	5	6	3	T3N0M0	0	RC + LND	T0N0	
8	76	M	1	1	5	4	5	3	T3N0M0	0	RC + LND	T0N0	
9	71	F	1	1	5	4	5	3	T3N0M0	2	RC + LND	T3N0	378
11	49	F	1	1	5	5	5	3	T2N0M0	2	RC + LND	T0	Other*
12	66	M	1	1	4	4	4	3	T2N0M0	2	Rad	—	269
13	58	M	1	1	5	5	5	3	T3N0M0	1	RC + LND	T2N1	507
15	66	F	0	1	6	5	6	3	T3N1M0	3	RC + LND	T3N2	293
18	56	M	1	1	6	6	6	2	T4N0M0	2	RC + LND	T3N2	264
19	57	M	1	1	6	5	5	3	T3N0M0	1	RC + LND	T0N0	
21	64	F	1	1	4	4	4	3	T3N0M0	2	RC + LND	T0	466
24	66	M	1	1	5	5	5	3	T3N0M0	1	RC + LND	T0 N1	
26	50	M	1	1	6	6	6	3	T3N0M0	1	Partial RC + Rad	T3	472
27	58	M	1	1	4	3	4	3	T3N2M0	2	RC	T0	
29	59	M	1	1	6	6	6	3	T3N0M0	ND	RC + LND	T3	
32	65	M	1	1	5	3	4	3	T3N0M0	1	RC + LND	T3	283
33	70	M	1	1	4	4	4	3	T3N1M0	0	1. CR 2. LND	N0	

The median age at time of TUR was 59 with the cohort consisting of 12 males (M) and 5 females (F), in keeping with the prevalence of BC. The number of TUR, BUF, PLS, UCP and USN samples obtained for each patient are presented. All patients with MIBC had high-grade (G2-3), locally advanced disease and opted for Radical Cystectomy (RC). Furthermore, 8/17 patients had early recurrence (median 336 days, ranging from 264 to 507). One patient (*) died shortly after surgery due to surgical complication and was thus excluded from further analysis involving correlations with early recurrence outcome. TUR and imaging stage information and final pathology are provided as per TNM criteria. CR – Chemoradiotherapy, Rad – Radiotherapy, LND – Lymph Node Dissection, ND – Not done.

We re-sequenced TUR samples to a median depth of 9600X for SNV analysis and a mean of 14.1 million sequencing reads per sample for CNA analysis. To detect mutDNA in liquid biopsy samples, we performed re-sequencing to a median depth of 7600X for SNV analysis and a mean of 13.6 million reads/sample for CNA analysis. We confirmed that there was no sample crossover between different patients by comparing single nucleotide polymorphism (SNP) profiles (Supplementary Figure 4).

### Detection of DNA alterations in TUR samples from MIBC patients undergoing NAC

**Figure 2:**
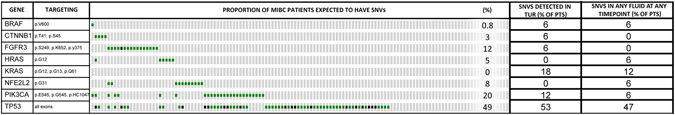
Genomic regions interrogated by TAm-Seq for SNV analysis. ^^^Specificity of TERT assays was poor due to constraints of targeting short amplicons in the repetitive TERT promoter region. Data resulting from these assays were therefore excluded from downstream analysis. Other than TERT, 90–100% of mutations reported in the above listed genes were covered by the panel. Alterations in these genes would capture 72% of alterations reported in MIBC patients. The prevalence of the mutations shown here are based upon data generated by the TCGA Research Network: http://cancergenome.nih.gov/. Green squares represent missense mutations and black squares represent truncating mutations^38, 39^. The number of patients (and percentage of patients) with SNVs detected in TUR and in any body fluid at any time-point are shown for each gene.

We performed TAm-Seq using a bladder-specific sequencing panel (Fig. 2 and methods) on all samples, for the analysis of SNVs, as previously described^25^. The initial panel design included primers targeting the promoter region of *TERT*, but the resulting short amplicons (<120 bp) did not perform well due to the repetitive sequences in this region and were excluded from further analysis. We detected a total of 22 SNVs across 12 of the 16 patients (Fig. 1C, Supplementary Table 1). The most frequent mutations detected were in *TP53* (14 mutations across 10 patients), followed by *KRAS* (3 mutations), then *PIK3CA* (2 mutations). One SNV each was also detected in the *BRAF*, *CTNNB1* and *FGFR3* genes. These findings agree with previous studies annotating the frequency of SNVs in MIBC^24, 26^. Though the numbers of SNVs detected in TUR samples varied amongst patients, there was no correlation with SNVs in TUR per patient and clinical outcome.

Furthermore, we developed an sWGS approach, adapted from published methods^27^, to assess CNAs in TUR. Gross genome wide CNAs were detected in all tumour specimens, including for those 5 patients in whom no SNVs were observed (Fig. 1C). Across the 16 TUR samples, we detected focal *CDKN2A loss* (*37*.*5%*), *E2F3/SOX4 gain* (*37*.*5%*), *PPARG gain* (*25*.*0%*), *YWHAZ gain* (*18*.*8%*), *CREBBP loss* (*12*.*5%*), *MYCL1 gain* (*12*.*5%*) and CNAs of other BC genes, as previously shown^24^.

### Comparison of genomic profiles in tumour and pre-NAC peripheral samples and correlation with response to NAC

We collected samples immediately prior to starting NAC, 1–2 months after TUR (Supplementary Table 1). Of the 12 patients with SNVs detected in TUR, one or more identical SNVs were also detected in 30.8% (4/12) 46.2% (5/12) and 46.2% (5/12) of pre-NAC plasma, UCP and USN samples respectively. Similarly, CNAs were observed in 25% (4/16), 53.3% (8/15) and 50% (8/16), and of pre-NAC plasma, UCP and USN samples of all patients with available samples. When a mutDNA CNA signal was detectable in peripheral fluids, the CNA profile subjectively matched the CNA profile of the corresponding TUR. Linear modeling of CNA profiles from each peripheral fluid, in which a CNA was called and the matched TUR resulted in a median adjusted R^2^ of 0.4990 (range 0.0011–0.9777) and is likely to represent the reduced prominence of CNA profiles in the peripheral samples, possibly due to higher levels of germ-line DNA in urine and plasma samples, due to successful removal of the bulk of disease by TUR, or due to spatial and temporal tumour heterogeneity (discussed below). When combining both methods, mutations (SNVs and CNAs) were detected in the first (pre-NAC) time-point in 58.8% (10/17) patients. MutDNA was present at the pre-NAC time-point in 35.3% (6/17), 47.1% (8/17) and 52.9% (9/17) of plasma, UCP and USN samples respectively.

MutDNA was detected in pre-NAC samples in 6 patients who showed complete or partial response and in 4 patients who showed no response or progression according to final pathology at radical cystectomy. 5 patients who responded and 2 patients who did not, showed no evidence of mutDNA at this time-point. Therefore, the detection of mutDNA in pre-NAC samples did not correlate with the early response to NAC in this sample set (Supplementary Table 2). 16/17 patients had diagnostic flexible cystoscopy following TUR, before NAC was commenced. On cystoscopy, 8/16 patients had obvious residual tumour present, and mutDNA was detected in the peripheral samples in 6/8 patients with residual tumour and in 1/3 patients with no obvious residual tumour. mutDNA was detected in a further 3/5 patients with equivocal cystoscopy findings. There was no correlation with patient age, sex or stage and the pathological response to NAC, nor the status of recurrence.

### Presence of mutDNA during NAC is associated with recurrence

The majority of mutDNA was detected in samples taken from patients who recurred after definitive therapy (Fig. 1C). 8 patients in our cohort recurred, with a median time to recurrence of 336 days (maximum 507 days) from initial TUR. One patient (patient 11), died due to a post-operative complication of radical cystectomy and was categorised as “other” and excluded from further analysis. Patients 8 and 29 developed new primary malignant melanoma and lung adenocarcinoma tumours at 882 and 175 days respectively during follow up. These patients were censored at the date of new tumour diagnosis to be recurrence free for the purposes of our analysis. Therefore, a total of 8 patients were recurrence free after a median follow up of 781 days after TUR (maximum 1008 days). Overall, 90 SNVs were detected in 219 mutant-time-point analyses, and 26 CNAs were detected in 54 samples, from the 8 patients who recurred (Fig. 1C). However, only 4 SNVs were detected in 193 mutant-time-point analyses and 4 CNAs were detected in 55 samples tested from the 8 non-recurring patients. Chi-Squared comparison at each time-point showed a significantly greater mutDNA detection rate in patients that recurred as compared to those that did not at time-points 1, 2, 3 and 5, (p = 0.0058 at T1, p = 0.0055 at T2, p = 0.0130 at T3 and p = 0.0272 at T5 after Bonferroni correction for multiple testing). Also, SNV AFs at each time-point were significantly higher in patients who recurred compared to those who did not (Kolmogorov-Smirnov, p < 0.022 at all time-points after correcting for multiple testing, Supplementary Figure 5).

To investigate potential utility of mutDNA analysis for the prediction of recurrence in patients with MIBC, we analysed the persistence of mutDNA, in peripheral samples taken at an early on-treatment time-point (i.e. just prior to the administration of the 2^nd^ NAC cycle). We utilised TAm-Seq to detect mutDNA at this time-point (CNA data were not available) and as such 5 patients were precluded from further analysis as SNVs were not detected in their TUR nor peripheral samples. Presence of mutDNA was defined as its detection in one or more sample type(s) at an AF, greater than 1/genomic equivalent copies inputted per reaction (our input threshold, raw data in Supplementary Table 5) and higher than 0.5% (above our technical threshold). This technical threshold was previously described at 2% for the identification, and 0.14% for the detection of SNVs by Forshew *et al*. in 2012^25^. Whilst, AUC curves (Supplementary Figure 6) show that a number of thresholds could have been used, we utilised a working threshold of 0.5% for our proof of principle study as this ensured 100% specificity, and meant that in buffy coat samples AFs at all genomic co-ordinates with non-reference calls were below our threshold, Supplementary Figure 7. Whilst not possible here, future studies could improve on this with more samples and employ training and validation sets to determine the optimum threshold.

**Figure 3:**
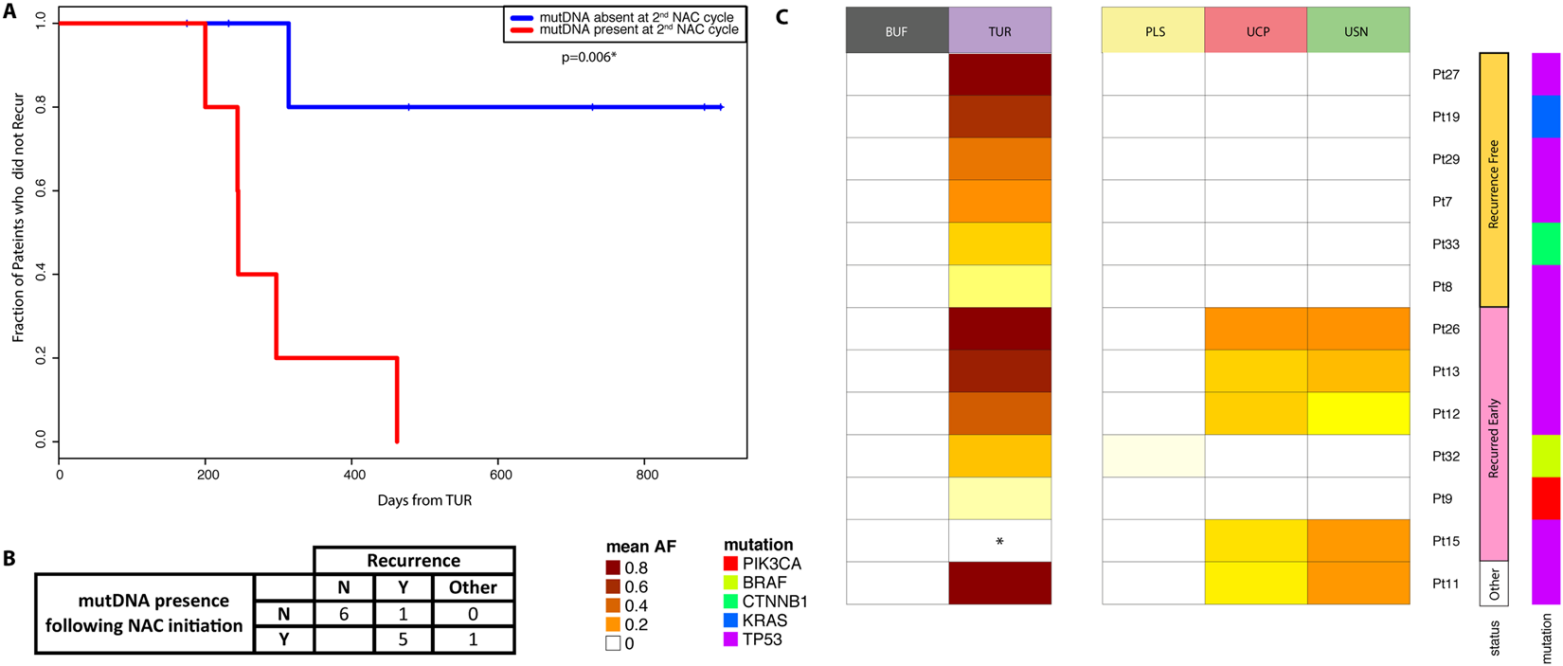
Presence of mutDNA at the 2^nd^ cycle of NAC predicts early recurrence in MIBC. (**A**) Kaplan-Meier curve depicting time to recurrence from initial TUR. We compared the rate of recurrence of patients with detectable mutDNA (red line) and undetectable mutDNA (blue line) in peripheral samples taken immediately prior to the 2^nd^ cycle of NAC (i.e. 2–3 weeks after the initiation of NAC). MutDNA was detected in 5/6 patients who recurred and in 0/6 recurrence free patients. Median time to recurrence in patients with detected mutDNA was 293 days while in patients with undetected mutDNA the recurrence rate was low. (**B**) Sensitivity and specificity for recurrence prediction. Overall sensitivity and specificity were 83.3% and 100% with positive predict value and negative predictive values of 100% and 85.7% respectively. One “other” patient was excluded from recurrence analysis due to post-operative death. (**C**) Heatmap comparing SNV maximum AF for each patient across all sample types and recurrence states at this time-point. Mutant allele fractions (mAFs) are represented by coloured cells ranging from white to scarlet as mAF increases (raw data in Supplementary Table 4). Patients are grouped by recurrence status. Generally, SNV mAFs are noticeably higher in USN and UCP as compared to PLS. There is a clear correlation between SNV mAF in peripheral samples and patient recurrence status.

According to these criteria, mutDNA was present at the 2^nd^ NAC cycle visit in 5 of the 6 patients that recurred, whereas it was not detected in any of the cases that did not recur, 83% sensitivity (95% CI: 36–100%) and 100% specificity (95% CI: 42–100%), (Fig. 3A and B). All of the patients with detected mutDNA at the 2^nd^ NAC cycle had disease recurrence, with a median time to recurrence of 293 days (Fig. 3), whereas patients in whom mutDNA was not detected had a low recurrence rate (p = 0.006 using log rank test Fig. 3A resulting in 100% positive predictive value and 85.7% negative predictive value, Fig. 3B). For the single patient (patient 9) who recurred despite not having detectable mutDNA at this time-point in any peripheral sample, the tumour had a *PIK3CA* E545K mutation that was present at an AF of only 0.7%. It is likely that this mutation represents a minor subclone of cells in the tumour and therefore may not be present in recurrent tumour. As a biomarker, mutDNA detection in samples taken at cycle 2 of NAC offered a median lead-time over radiological detection of recurrence of 243 days (range 182–455 days) in our data. This association was primarily driven by detection of *TP53* SNVs in the urinary samples (Supplementary Figure 8), where 4/5 patients that recurred had mutDNA while only 1/5 patients had a *BRAF* SNV detected in their plasma (Fig. 3C, raw data in Supplementary Table 4).

### Comparison of peripheral sample types reveals that UCP and USN are enriched in mutDNA as compared to plasma

**Figure 4:**
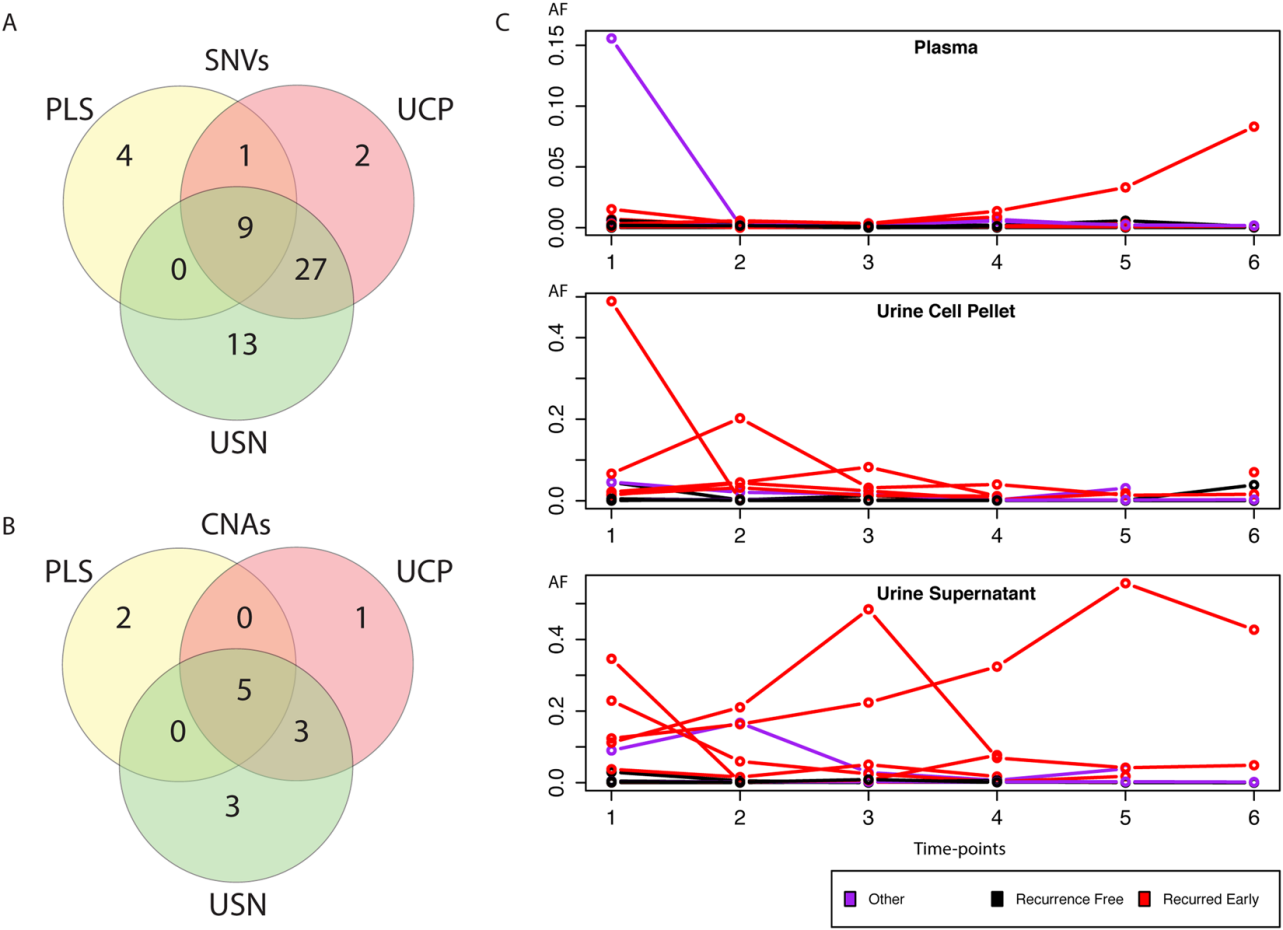
Longitudinal analysis of peripheral samples for non-invasive detection of tumour profiles in MIBC. (**A**+**B**) Venn diagrams demonstrating that more SNVs (**A**) and CNAs (**B**) were detected in the urine, as compared to the plasma samples. The number of times SNVs or CNAs were detected in peripheral samples per time-point (where all three peripheral samples were collected) were depicted as Venn diagrams. 52 out of 56 SNVs and 12 out of 14 CNAs were detected in urinary samples. However for SNVs, 4 mutations/time-points were detected only in PLS, 2 in UCP and 13 in USN. For CNAs 2 mutations/time-points were detected only in PLS, 1 in UCP and 3 in USN, confirming that multiple sample analysis can improve mutDNA detection in MIBC. (**C**) Maximum mutDNA AF during NAC demonstrates differing kinetics in PLS, UCP and USN. Three plots depict the maximum SNV AF at each time-point in PLS, UCP and USN samples for 13 patients with detected SNVs. There are clear differences in the AF kinetics between the peripheral sample types. Generally levels are low in PLS while AFs rise and fall dynamically in urinary specimens. For most patients, mAFs are low during NAC, however, mAFs that were considerably higher than the 0.005 AF detection threshold were found in patients that recurred.

To identify the most informative peripheral sample type for mutDNA analysis in MIBC, we analysed each SNV detected at a single time-point as an independent variable. At 86 time-points all 3 sample-types were drawn simultaneously, with each of the 31 mutations being detected in at least one of the peripheral samples in this group. SNVs were detected most frequently in USN (34.5%, 49/142), UCP (27.5%, 39/142) and lastly plasma (9.9%, 14/142). Analysing the mutant AFs without imposing detection criteria^25^, AFs were not statistically different between USN and UCP but were higher in UCP and USN when compared to plasma (both with p < 0.0001 by Kruskall Wallis and Dunn testing, Fig. 4A and Supplementary Figure 9). However, no single peripheral sample type captured all of the SNVs that were detected across all the samples together, and for each sample type there were SNVs that were unique to it. Indeed, 4 of the events (individual SNVs detected in individual time-points) were detected only in plasma, 2 only in UCP, and 13 only in USN. Similarly, CNA analysis was conducted for the first and last time-points, where all three peripheral samples were analysed. When CNAs were detected, they were frequently found in all three peripheral sample types. However, more CNAs were detected in USN and UCP than plasma but the small numbers precluded statistical interpretation (Fig. 4B).

We also compared whether SNVs and CNAs co-occurred and whether there was a relationship between them. In pre-NAC peripheral samples, though detection rates for SNVs and CNAs were similar, SNVs were not always detected in the same samples as CNAs. Considering all samples, there was generally a greater representation of CNAs in samples with higher SNV AFs. However, there were examples of CNA detection where SNV AFs were low or were absent altogether (Fig. 1C and Supplementary Figure 10). This was apparent in all peripheral sample types. This may be due to the ability of sWGS to interrogate a greater breadth of the genome than targeted re-sequencing approaches or due to a difference in tumour biology whereby some tumours are CNA driven with an absence of SNVs. Our data demonstrates that sampling multiple body fluids using complementary techniques allows for more complete assessment of mutDNA.

### Longitudinal analysis of mutDNA in peripheral samples of patients with MIBC

We applied TAm-Seq to analyse recurrent bladder cancer associated genetic events in serial plasma, UCP and USN peripheral samples (taken from NAC initiation through to its completion) to assess trends in mutDNA. Peripheral samples were collected longitudinally from each patient over a median of 83 days (46–118 days, median of 15 samples/patient). CNA analysis was initially applied to the first and final time-points for all patients across all peripheral sample types. For both SNV and CNA dynamics, the overall trend was a reduction in mutDNA over time during NAC (Figure 4C and Supplementary Figures 11–14). However, there were examples of persisting mutDNA, particularly in the urinary samples. Furthermore, there were patients who showed variation in mutDNA AFs over time.

### Urinary mutDNA demonstrates tumour evolution on-therapy

As opposed to assays targeting mutations detected in matched tumour samples, combined use of our disease specific assay and sWGS allowed the detection of *de novo* mutations. To determine additional examples of tumour evolution we performed sWGS analysis of samples taken between the first and last time-points in selected patients based on high SNV levels or interesting CNA profiles at the first time-point (Fig. 1C). In 5 patients there was evidence of dynamic tumour evolution during NAC, highlighting the strength of studying mutDNA in peripheral fluids as an alternative to traditional biopsy approaches^25, 28^. All *de novo* mutations were detected in the urinary specimens and were not detected in the initial tumour specimen. There was no correlation between the detection of these private mutations and clinical outcome.

In patient 12, whilst mutDNA levels in plasma fell quickly following the initiation of NAC, urinary mutDNA levels remained high and reached a peak at 85 days after TUR. Additionally, a new nonsense mutation of *TP53* (W53*) was identified in urinary samples at this time-point (3.6% in UCP and 0.7% in USN), suggesting the emergence of a new clone (Supplementary Figure 15a). Similarly overall CNA levels rose in parallel with SNV AFs and also demonstrated the emergence of a new CNA profile (including a novel focal amplification of *GRIN2A*)^29, 30^. The short-term persistence of this clone was confirmed by sWGS analysis of additional urine samples from this patient (Supplementary Figure 15b). Levels of all mutations ultimately fell, though some were detected at the last sample taken during NAC. Although a good response to NAC was initially reported for this patient, he developed brain metastases approximately 2 months following radical radiotherapy to the bladder.

**Figure 5:**
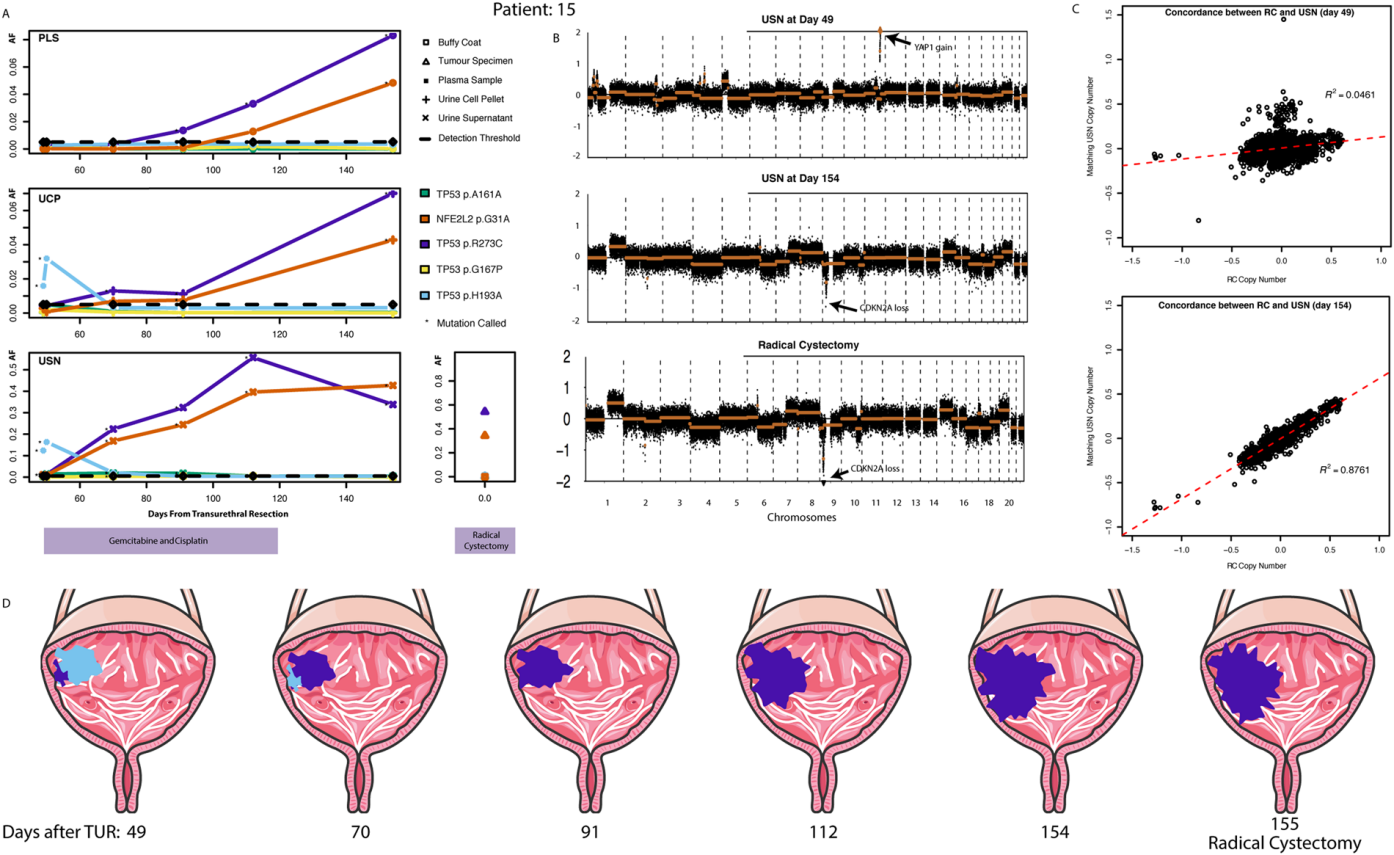
mutDNA demonstrates on-therapy tumour evolution. (**A**) SNV plots for patient 15 demonstrate tumour evolution. SNV analysis of pre-NAC urinary samples revealed a *de novo TP53* H193A mutation (light blue), whilst *TP53* R273C (purple) and *NFE2L2* G31A (orange) SNVs were only observed at low AFs in USN samples (1% and 1.1% respectively). During NAC, the clone containing *TP53* R273C and *NFE2L2* G31A SNVs appears to grow considerably, whilst the *TP53* H193A SNV containing clone recedes to become undetectable at later time-points. This profile was mirrored by the cystectomy sample. (**B**) CNA profiles demonstrate tumour evolution. Marked CNA changes were observed in urine sample at pre-NAC (including *YAP1* focal amplification). This CNA profile differed from that seen in later time-points. At time-point 6, the CNA profile resembles one seen at cystectomy. (**C**) Concordance of cystectomy and USN CNA profiles during NAC. We generated a linear model by fitting autosomal 1 Mb bin read-counts in the cystectomy sample against those in peripheral samples. Initial USN CNA profiles are discordant with the cystectomy sample (R^2^ = 0.0461). Subsequently a concordant CNA profile emerges (R^2^ = 0.8760), mirroring the SNV results. D. Longitudinal mutDNA analysis suggests on-therapy tumour evolution. We used the changing SNV and CNA profiles to suggest a clonal evolution paradigm in patient 15. (Images adapted from Servier Medical Art).

Patient 15 had T3N1 disease that recurred 138 days after cystectomy. The SNV and CNA profiles observed in her urine specimens suggested tumour evolution in response to surgery and/or NAC. Specifically, a clone characterised by a *TP53* H193A SNV and a focal amplification of *YAP1* was dominant at the pre-NAC time point. This sample also carried *TP53* R273C and *NFE2L2* G31A SNVs but they were detectable at low AFs (Fig. 5A and B) and likely represent a minor clone. During NAC, the initially dominant (presumably NAC sensitive) *TP53* H193A, containing clone receded, whilst the minor clone containing *TP53* R273C and *NFE2L2* G31A SNVs and a focal loss of *CDKN2A* became dominant in all peripheral fluid samples. Radical cystectomy was carried out 155 days after TUR and 106 days after the initiation of NAC. We obtained DNA from the cystectomy sample and carried out TAm-Seq and sWGS on it. Of note, we found that the clone containing *CDKN2A* loss and *TP53* p.R273C and *NFE2L2* p.G31A SNVs was present at high levels, whilst there was no evidence of the clone containing *YAP1* gain and the *TP53* p.H193A SNV (Fig. 5A and B). The similarity between peripheral samples obtained during NAC and the subsequent cystectomy samples was confirmed by modelling the linear relationship between CNA profiles of these samples (Fig. 5C). We also carried out additional sWGS of intermediate time-points for this patient and confirmed gradual loss of the pre-NAC CNA profile below our detection threshold. Together this data points to ‘on-therapy’ tumour evolution under the selective pressures of NAC, as indicated by apparent changes in the dominant clone and its respective AF (Fig. 5D). Whilst most noticeable in USN, this evolution was also evident in matched UCP samples, albeit at lower levels. Plasma mutDNA analysis alone would not have demonstrated evolution in this case, although it did reveal the emergence of the later clone containing *CDKN2A* loss and *TP53* p.R273C and *NFE2L2* p.G31A SNVs, Supplementary Figure 16. This is possibly due to low mutDNA levels at the early time-points, which may in turn be due to spatial differences in the clones resulting in different representation of shed DNA in the plasma and urine.

### MutDNA analysis can be valuable in multiple disease settings of urothelial cancer

To explore the applicability of mutDNA detection in other urothelial cancer settings we used the same methods to analyse mutDNA levels in 3 additional patients: patient 1 had ureteric urothelial cell cancer (UCC), patient 10 had metastatic BC and patient 30 had renal pelvis UCC (Supplementary Table 3). For patient 1, SNVs in *TP53* and *KRAS* that were identified in tumour DNA, were detected at low levels (AFs < 5%) in different peripheral samples at different time-points during treatment (Supplementary Figure 17). For patient 10, the CNA profile observed in the tumour (including focal loss of *CDKN2A* and *CREBBP*) was observed in all peripheral sample types taken prior to starting chemotherapy, albeit at lower levels in plasma. MutDNA levels, as inferred by the CNA profile, gradually decreased to below the detection threshold of sWGS, first in plasma, and then in UCP and USN during treatment (Supplementary Figure 17). For patient 30, the tumour biopsy did not yield sufficient material for DNA extraction. Nonetheless we detected SNVs in *TP53* and *PIK3CA*, as well as CNAs (including focal amplifications of *MYCL1*, *E2F3/SOX4* and *PPARG*, and focal loss of *CDKN2A*) in all peripheral samples. SNVs were detected at consistently high AFs in the urinary specimens but at moderate levels in the plasma. Matching CNA profiles were seen only in the urinary samples, where they persisted between the two time-points analysed (Supplementary Figure 17). These preliminary findings suggest that analysis of mutDNA in body fluids may be useful for disease monitoring in multiple urothelial cancer settings.

## Discussion

MutDNA analysis has been shown to have translational potential in many solid cancers^31^. MutDNA has been studied in plasma but little data has been presented on the analysis of mutDNA in urine. In our cohort of MIBC patients we confirm the presence of SNVs and CNAs previously reported in BC^24, 26^. We confirm that mutations found in TUR samples are detectable in both the plasma and urine of BC patients. Our data, based upon the analysis of 86 time-points, demonstrate that mutDNA is detected more frequently and at higher levels in urine (both USN and UCP) and agrees with previous work^23^. Importantly however, in some cases mutDNA was only detected in one individual sample type. These private mutations could represent local anatomy or biology. For example, one could speculate that mutations private to plasma represent tumour clones situated deeper in muscle and/or closer to the vasculature. This analysis may be important as such clones could have different clinical outcomes. In this study, the observation of such plasma-specific mutations did not associate with response to NAC or early recurrence, though the number of patients was small. Nonetheless, our data indicates an advantage in assessing both urine (UCP & USN) and plasma for the comprehensive analysis of mutDNA in MIBC. Additionally, the observation that urinary AFs were higher than plasma AFs, suggests that the presence of mutDNA in urinary samples is due to direct tumour shedding. Beyond BC, this study confirms the role of sampling peripheral fluids in close proximity to diseased organs in order to improve the sensitivity of the detection of mutDNA^32, 33^.

Of potential clinical significance, we found that detection of mutDNA at the second cycle of NAC (2–3 weeks after initiation of therapy, depending on the specific regimen) using our methods was indicative of early disease recurrence, with a sensitivity of 83% and specificity of 100%. We acknowledge that we have studied a small number of cases and with a relatively short follow-up period (possibly resulting in recurrence events being missed). However, it is noteworthy that MIBC often recurs within 2 years^34^ and our recurrence rates are comparable with published data^35^. Our proof of principle study therefore encourages further large-scale investigation. In our series, the single false negative case was likely due to the narrow focus of our bladder-specific panel, since we only detected a *PIK3CA* mutation in the TUR sample at low AF, which likely represents a minor sub-clone (similar to those described in patients 12 and 15). Analysis of additional targets, through the use of an expanded panel for targeted sequencing or by capture-based strategies (as has been used in other cancer types)^28, 36^, may have identified alternative cancer pathway mutations in this patient. Whilst we attempted to overcome this by interrogating the wider cancer genome through CNA profiling^24^, we note that CNA analysis is limited to detecting mutDNA at ~5% mutant:wild type AF ratio^27^. Alternatively, by applying only *TP53* detection to the USN of the 9 patients who had *TP53* mutations in their TUR, a focussed *TP53* urinary assay would have detected all 4 patients that recurred (Supplementary Figure 8).

**Figure 6:**
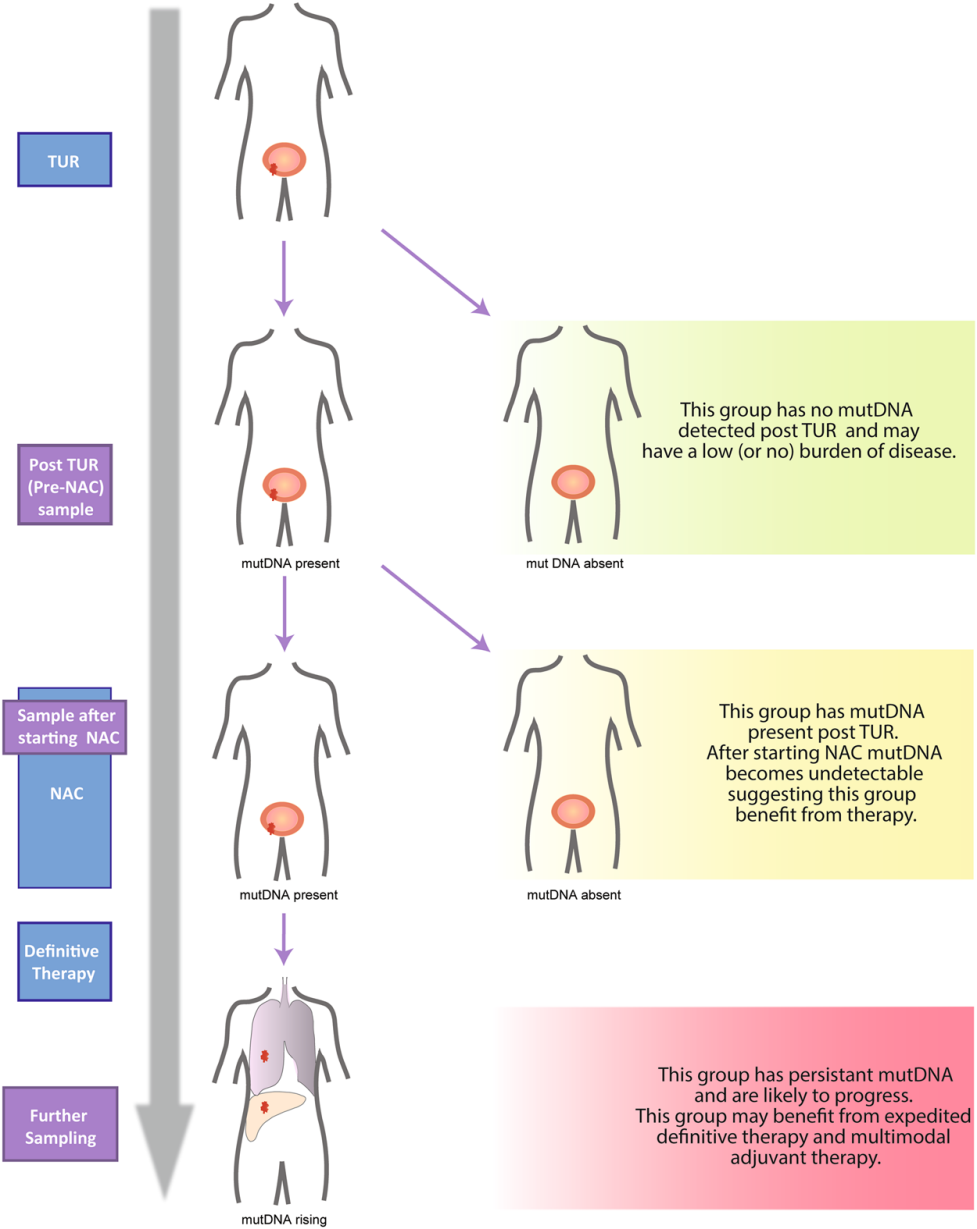
MutDNA analysis may help stratify patients with MIBC in the future. Based on our data, we have generated a model by which a patient’s outcome can be predicted by mutDNA status before and during NAC. Specifically, mutDNA status may be used to stratify patients into 3 groups. Firstly, patients with undetectable mutDNA pre-NAC and during NAC, have a low (or no) burden of disease (green). Secondly, patients with detected mutDNA pre-NAC but with negative samples at the 2^nd^ NAC cycle are likely to benefit from their NAC and subsequent definitive therapy (yellow). Finally, patients with mutDNA detected before and during NAC are unlikely to benefit from continued NAC and often progress (red). These patients should be considered for expedited definitive treatment or alternative treatments (e.g. targeted therapy or immune checkpoint inhibition).

If confirmed by larger studies, mutDNA detection in pre-NAC samples and in samples taken before the 2^nd^ NAC cycle may be used to stratify patients into 3 groups (Fig. 6). Firstly, those that are negative at both time-points may have a low (or no) burden of disease after TUR. Secondly, those that have a positive pre-NAC sample but negative samples at the 2^nd^ NAC cycle are benefiting from their NAC and subsequent definitive therapy. The final group of patients has detectable mutDNA at both time-points and often progresses. Patients in this group are not benefiting from NAC and should be considered for expedited definitive therapy or alternative systemic therapy (e.g. targeted therapy or immune checkpoint inhibition). The specific mutations arising (or persisting) during therapy may inform further therapeutic strategy. Analysis of the presence of mutDNA in samples from later time-points was also informative but, would be less clinically useful. An early decision on continuation of NAC would prevent administration of multiple cycles of toxic therapy that is not benefiting the patient (Fig. 1C).

We provide the first description of tumour evolution through *de novo* mutation detection in urinary specimens. We hypothesise that the changes in the mutational profiles observed in these patients represent tumour evolution under the selective forces of surgery and NAC. This is exemplified by patient 15. It is likely that multiple clones were present initially, with analysis of initial urinary samples highlighting a dominant somatic profile consisting of *TP53* H193A and *YAP1* gain. Initiation of NAC allowed for the apparent emergence of a distinct (presumably NAC resistant) tumour clone, containing, *TP53* R273C, *NFE2L2* G31A and *CDKN2A* loss. Meanwhile the clone containing *TP53* H193A and *YAP1* gain mutations appears to respond well to NAC and recedes. Our data emphasises the presence of multiple concomitant tumour clones and, the importance of monitoring mutDNA with an entire sequencing panel (e.g. one targeting all of *TP53*), rather than relying on the detection of known mutations from matched tumour samples. In addition, though we cannot rule out the effects of tumour heterogeneity in biasing our radical cystectomy sample analysis, the equivalent longitudinal data from peripheral fluid samples suggests that a combined body fluid sampling method is capable of overcoming the effects of spatially distinct clones. Overall, our data demonstrates the strength and potential of mutDNA profiling for non-invasive monitoring of clonal dynamics throughout therapy.

Our proof of principle study has several weaknesses. Patient numbers were small and thus firm clinical conclusions cannot be made. However, by comprehensively studying several biological samples per patient at various time-points during NAC, we were able to establish a detailed overview of mutDNA in BC and generate specific hypotheses that can now be tested in prospective, sufficiently-powered, clinical studies. In addition, although the presence of mutDNA at the second cycle of NAC was associated with clinical outcome, we found instances where patients with favorable outcome had mutDNA detected in samples at later time-points. At this point, it is unclear whether these findings are clinically meaningful. However, it is clear that patients with a favorable outcome tend to lose detectable presence of mutDNA (Fig. 1C). Finally, the detection methods chosen have limitations in panel size, sequence depth and sensitivity. Although improvement is possible, we believe these represent a reasonable trade-off in comparison with methods like digital PCR (high coverage but few mutations analysed) and capture-based NGS assays (large gene panels but coverage limited by sequencing costs).

Together with previous work, we have demonstrated the utility of urinary mutDNA analysis across the full spectrum of bladder cancer. Using multiple sample sources and complementary genetic techniques we have provided a more rigorous analysis of mutDNA. Furthermore, despite the small numbers of patients, our study highlights the important role that mutDNA analysis can have in predicting aggressive disease in MIBC and could offer an opportunity for patients to consider expedited definitive therapy or alternative regimens.

## Methods

### Sample Collection

Approval according to national guidelines was obtained from the NKI Translational Research Board (N13KCM/CFMPB250) for the longitudinal analysis of mutDNA in MIBC. All patients gave informed consent to participate in this study. All experiments were approved by the Cancer Research UK-Cambridge Institute and NKI. Experiments were performed in accordance with relevant guidelines and regulations. Formalin-fixed paraffin-embedded (FFPE) tumour blocks from the TUR samples were collected from referring hospitals. Slides were cut from FFPE blocks and used for Haematoxylin and Eosin (H&E) staining and DNA extraction. H&E stains were evaluated to identify areas with >50% tumour cells, from which DNA was extracted using the QIAamp DNA mini kit (Qiagen, Germany). Urine and blood samples were collected prior to each chemotherapy session and were processed as follows; 10 ml of peripheral blood was drawn into K2-EDTA haematology tubes and centrifuged at 380 g for 20 mins. The buffy coat layer was carefully transferred before the remaining plasma was aliquoted and spun at 20,000 g for 10 mins. We found that 3 ml urine samples were optimally processed by immediate addition of 0.5 M EDTA with subsequent centrifugation at 380 g for 20 mins and aliquoting the urine supernatant, leaving the cell pellet (Supplementary Figure 1). UCP was re-suspended in 1 ml of PBS. All peripheral fluids were processed within 6 hours and stored at −80 °C.

Furthermore, we found that DNA extraction from 3 ml of urine supernatant by Circulating Nucleic Acid (Qiagen) and Urine DNA Isolation, Slurry Format (Norgen, Canada) kits were equally suitable, Supplementary Figure 1. We opted to extract DNA from TUR, BUF, UCP and plasma specimens with QIAamp FFPE Tissue, DNeasy, DNA Mini Blood Mini and, Circulating Nucleic Acid (Qiagen) or Qiasymphony respectively. Samples were split and DNA extracted by manual and Qiasymphony extraction. Comparison of resulting copies/ml and mAFs showed no difference between the two methods (Supplementary Figure 2).

DNA extracted from BUF and UCP samples were subjected to mechanical shearing using either a Covaris S220 or LE220 (Covaris, USA). TUR was also sheared using the same protocol due to improved FFPE preservation methods leading to longer DNA fragment sizes^37^. Recommended parameters were used to shear fragments to an average fragment length of 140–180 bp. Successful shearing was confirmed by running 1 μl of DNA on a High-Sensitivity Bioanalyser gel (Agilent, USA).

### TAm-Seq

Tagged-AMplicon Sequencing (TAm-Seq) primers were designed to assess the single nucleotide variant (SNV) status for hotspots or entire coding regions of 8 genes commonly mutated in bladder cancer, based on recent WGS studies^24^ and the COSMIC database^26^ (Fig. 2). Genes were incorporated into the panel based on the frequency of mutation in WGS studies of BC. Primer details are available on request. DNA input amounts for TAm-Seq are provided in Supplementary Table 5. TAm-Seq libraries were prepared as previously described^25^. Libraries were sequenced using an Illumina HiSeq 2500 (Illumina, USA).

### sWGS

Libraries were prepared from 10ng of plasma, USN, and sheared TUR, BUF and UCP DNA using ThruPLEX Plasma-Seq (Rubicon Genomics, USA). Briefly, end repair and ‘A-tailing’ of fragment ends preceded the ligation of truncated Illumina sequencer compatible adapters to fragment ends. Thermocycling of libraries completed the adapters through the addition of sample specific index sequences, and was performed as described in the Plasma-Seq protocol, using 8 (TUR and BUF) or 8–14 (plasma, UCP and USN) amplification cycles depending on subsequent DNA concentration as estimated by inspection of bioanalyser traces. Following amplification, libraries were cleaned with Agencourt AMPure XP beads (Beckman Coulter, USA) at a 1:1 (v/v) ratio and eluted in 30 μl nuclease-free water. Successful library preparation was confirmed using a High-Sensitivity Bioanalyser gel and libraries were quantified using SYBR green based qPCR (Kapa Biosystems, USA). Libraries were pooled in an equimolar fashion and 125 bp paired end sequencing was performed (to give a mean of 14.2 million reads per sample) using an Illumina HiSeq 2500 or HiSeq 4000.

### Mutation Calling Criteria

TAm-Seq sequencing reads were aligned using BWA and SNVs were detected using proprietary SNV calling software, the principles of which were described previously^25^. All mutation calling was performed blinded to the patient outcome. Patient-specific mutation calls were used to determine mutant AFs for any time-point from the 13 patients with detected SNVs, with thresholds defined by the highest of mutDNA AF > 0.5% (technical threshold, see Results Section) or 1/genomic equivalent copies inputted (sample threshold, raw data in Supplementary Table 5). Bases that contained a mutant call at a frequency below this threshold were not used for further analysis though AF’s were retained for interpreting longitudinal mutDNA dynamics. NGS data were analysed using the R statistical software package^40^.

For sWGS analysis, sequence data was analysed using an ‘in-house’ pipeline that briefly consists of the following; paired-end sequence reads were aligned to the human reference genome (GRCh37) using BWA (version 0.7.13)^41^ after removing any contaminant adapter sequences. SAMtools (version 1.3.1)^42^ was used to convert files to BAM format. PCR and optical duplicates were marked using Picard-Tools’ (version 2.2.4) ‘MarkDuplicates’ feature and these were excluded from downstream analysis along with reads of low mapping quality and supplementary alignments.

CNA calling was performed in R^43^ using the QDNAseq pipeline^44^. Briefly, sequence reads were allocated into equally sized (here 1 Mb and 50 kb) non-overlapping bins throughout the length of the genome. Read counts in each bin were corrected to account for sequence GC content and mappability, and bins corresponding to previously ‘blacklisted’ (ENCODE) and manually blacklisted regions were excluded from downstream analysis. Within the QDNAseq package, bins were segmented using the ‘Circular Binary Segmentation’ algorithm^45^ and significantly ‘amplified’ or ‘lost’ regions were called using CGHcall^46^ – regions were called in peripheral fluids independent of the calls from the corresponding TUR sample.

We compared overall levels of copy number imbalance across the length of the genome in our samples by calculating a ‘genome-wide imbalance score’. To generate this value, log2 adjusted read counts in a given 1 Mb bin were compared against the equivalent value in a control sample. This control sample, which consisted of pooled sWGS data from 8 buffy-coat samples, was used for all pairwise comparisons. A linear model was fitted against all autosomal bin values of the test sample vs. the control sample and the squared sum of residuals of this fit was calculated. To overcome inherent noise surrounding baseline (i.e. copy number neutral) in sWGS data, we only considered the sum of the 5% most extreme residual values to represent the ‘genome-wide imbalance score’.

The data that support the findings of this study are available from the corresponding author upon request.

### Statistical Inferences

Statistical conclusions were impacted due to the proof of principle nature of the study. Where applicable we employed the following statistical analyses: To compare raw AF’s of patients who did and did not recur, these two populations were plotted in the form of an empirical cumulative distribution function and assessed by applying the Kolmogorov–Smirnov test. Analyses of correlation between AFs at the 2^nd^ NAC cycle and recurrence was performed using the “OIsurv” package in R^47^. Survival curves were generated using the ‘survfit’ function, which uses the Logrank test to compare differences (in the presence of censoring)^47^. Exact binomial confidence limits for sensitivity and specificity are calculated using the ‘epiR’ package in R^48^. The SNV AFs in different sample types was compared using a Kruskal-Wallis rank sum test after testing for normality. sWGS profiles were compared by applying linear-regression modelling to the log ratios of two samples and adjusted R^2^ values were generated using the linear model function in R.

## Electronic supplementary material

Supplementary Information

Supplementary Tables
