# Supplementary material for: Association Of Plasma And Urinary Mutant DNA With Clinical Outcomes In Muscle Invasive Bladder Cancer
Source: Sci Rep. 2017 Jul 17;7:5554. doi: 10.1038/s41598-017-05623-3 (PMC5514073; doi:10.1038/s41598-017-05623-3)
Supplement: Supplementary file 1 — Supplementary Information [file 41598_2017_5623_MOESM1_ESM.doc]

## Title:

Association Of Plasma And Urinary Mutant DNA With Clinical Outcomes In Muscle Invasive Bladder Cancer.

## Authors:

KM Patel^1,2,3♯^, KE van der Vos^4♯^, CG Smith^1,2♯^, F Mouliere^1,2^, D Tsui^1,2^, J Morris^1,2^, D Chandrananda^1,2^, F Marass^1,2^, D van den Broek^5^, DE Neal^1,6^, VJ Gnanapragasam^3^, T Forshew^1,2,7^, BW van Rhijn^8^, CE Massie^1,2^, N Rosenfeld^1,2^*, MS van der Heijden^4,9^*.

^♯^These authors contributed equally.

* These authors are joint senior supervisors

# Supplementary Material:

Supplementary Figure 1: Optimisation of urinary DNA extraction methods. Urine samples from 7 healthy volunteers were used to optimise urinary supernatant processing and extraction procedures (ethical approval LREC 03/018). Urine from volunteers was aliquoted into 3ml replicates for testing. All extracted DNA was analysed for yield using dPCR of a 97bp amplicon targeting the *RPP30* gene. Separate analysis showed little difference in yield between assays targeting 65bp and 97bp *RPP30* amplicons (performed in singleplex, data not shown). A. Box plots demonstrating that QIagen CNA and Norgen slurry kits achieved the highest yield of DNA from aliquoted urinary samples. Four commercially available DNA extraction kits with specific protocols for urine supernatant DNA extraction were tested. Shapiro Wilk testing demonstrated a non-Gaussian distribution for samples extracted by Norgen (W= 0.8141, p= 0.0042) and Qiagen kits (W= 0.7414, p= 0.0005). Guassian distributions were suggested for samples extracted by SnoMag (W= 0.9027, p= 0.3474) and NeoGene Star kits (W= 0.96173, p= 0.82). Due to the comparison of data sets with mixed distributions, non-parametric analysis was used to determine statistical significance of differing yields. Kruskall Wallis demonstrated statistically significant differences for the yield obtained by the extraction kits tested. Dunn testing demonstrated statistically higher yield from both Qiagen and Norgen extraction when compared with SnoMag (p=0.0028 and p=0.0088). B. Cell-free DNA levels are higher in urine samples processed without a centrifugation step. The increased cell-free DNA levels seen in the non-centrifuged samples, is likely to be derived from lysed cells. We utilised centrifugation to process USN samples separately from UCP samples as opposed to using whole urine. This was due to our expectation that UCP lysis would release large amount of wild-type DNA and dilute the mutDNA signal, based on the established insensitivity of urinary cytological analysis for BC detection. Shapiro Wilk testing revealed non-Gaussian distribution for the distributions (W= 0.38235, p= 2.564xe^-11^ for centrifuged samples and W= 0.47213, p= 3.123xe^-10^ for non-centrifuged samples). Kruskall-Wallis testing demonstrated a significant difference between the 2 data sets (p= 1.47xe^-05^). C. 0.5M EDTA improves cell-free DNA yield. Shapiro Wilk testing revealed non-Gaussian distribution for EDTA samples (W = 0.28299, p = 6.526xe^-12^) and for samples without EDTA (W = 0.39083, p = 2.176xe^-11^). Kruskall-Wallis testing did not show a significant difference between the 2 data sets (p= 0.0809). D. Reduced time to processing of fresh urine improved cfDNA yield. Shapiro Wilk testing revealed non-Gaussian distribution for samples processed at 1 hour (W= 0.40042, p= 2.881xe^-09^) for samples processed at 6 hours (W= 0.36372, p= 3.39xe^-09^) and for samples processed at 48 hours (W= 0.27938, p = 1.178xe^-09^). Friedman testing revealed statistically significant differences between the cfDNA amounts obtained with different processing times (p=0.0121). To allow for clinical utility, we endeavoured to limit the time taken to process a urine sample was to 6 hours during our clinical collections.

Supplementary Figure 2: Concordance of AFs in DNA from samples extracted by QIAsymphony and Circulating Nucleic Acid methods. Duplicate 3ml aliquots of 9 USN samples were paired. One aliquot was extracted using QIamp Circulating Nucleic Acid kit whilst its partner was extracted using QIasymphony (QIagen). A. Scatter plot of mAFs detected from replicate aliquots extracted by QIAsymphony and Circulating Nucleic Acid kits. TAm-Seq was used to estimate the mutDNA load for each sample. The line model is represented by a grey dotted line (slope = 0.847, adjusted R^2^ = 0.8942, p= 6.358xe^-12^). B. Scatter plot of total amplifiable copies detected from replicate aliquots extracted by QIAsymphony and Circulating Nucleic Acid kits. dPCR demonstrated no difference in amplifiable copies per ml of USN. The linear model is represented by a grey dotted line (slope = 0.778, adjusted R^2^= 0.5241, p= 0.0166).

Supplementary Figure 3: Amount of DNA (genome equivalent copies/ml) extracted from peripheral samples. Shapiro Wilk testing demonstrated non-Gaussian distributions for DNA extracted from plasma (W= 0.48227, p< 2.2xe^-16^), UCP (W= 0.65576, p= 8.183xe^-13^) and USN (W= 0.48084, p= 2.323xe^-16^) (W= 0.41295, p< 2.2xe^-16^). Kruskall Wallis testing demonstrated significant differences between the populations (p< 2.2xe^-16^). Dunn testing revealed that UCP samples had a statistically significant higher number of DNA copies/ml of fluid when compared with plasma (p<0.0001) and USN (p<0.0001). There was also a statistically significant difference between extracted DNA copies/ml from USN and PLS (p=0.0237). The respective medians and ranges in copies/ml for PLS, UCP and USN were; 3,550 (769-79,200) 61,613 (981–937,000), and 5,870 (101–236,000).

Supplementary Figure 4: SNP analysis demonstrating concordance of SNP genotypes for samples taken from the same patient. The analysis of 8 SNPs (that were included in our TAm-Seq panel) revealed that most patients have unique SNP profiles. Manual clustering of SNP profiles according to patient ID showed that SNP profiles from samples from an individual patient match other samples taken from the same patient and differ from those taken from other patients.

Supplementary Figure 5: Empirical Cumulative Distribution Function plot of SNV AFs for all samples at each time-point from patients who recurred (red) and those who did not (black). Each time-point was analysed independently. Kolmogorov-Smirnov testing was used to compare SNV AFs between recurrence and non-recurrence groups, with Bonferroni correction for multiple testing.

Supplementary Figure 6: AUC plots showing the trade off between sensitivity and specificity at various threshold cut-offs for prediction of recurrence using data from samples obtained immediately prior to the 2^nd^ cycle of NAC. Circles represent values obtained when using our threshold of 0.5% AF for mutation calling.

Supplementary Figure 7: Buffy coat AFs at TAm-Seq called locations with technical threshold plotted as 0.5%

Supplementary Figure 8: Kaplan-Meier curve depicting time to recurrence from initial TUR in a subset of 9 patients who had *TP53* SNVs detected in their TUR sample. All 4 patients who had *TP53* SNVs detected in their USN at the 2^nd^ cycle of NAC (red line) recurred, with a median time of 271 days. The 5 patients who did not have a *TP53* SNV detected in their USN at the 2^nd^ cycle of NAC (blue line) did not recur. When applied to MIBC patients with *TP53* SNVs detected in TUR specimens, the assay had a sensitivity and specificity of 100% for prediction of early recurrence.

Supplementary Figure 9: Allele Fractions of samples taken simultaneously. 86 time-points had paired sampling of all 3 peripheral sample types. In 64 cases a mutation was detected in one sample type, this and its paired sample AFs are plotted above with median AFs. The dashed line represents our technical threshold value of 0.5% AF. Shapiro Wilk testing for normality demonstrated a non-parametric distribution (W= 0.5087, p< 2.2xe^-16^). Kruskall Wallis testing revealed significant differences between the AF populations (p= 2.845xe^-12^), with post-hoc Dunn testing demonstrating significant differences between mutDNA AFs from both urinary sample types when compared to the plasma AFs, but no difference between UCP and USN mutDNA AFs.

Supplementary Figure 10: Waterfall plot depicting the relationship between CNAs and SNV AF. To determine whether there was a relationship between the detection of CNAs and the AF of called SNVs (when both data-sets were available), data were plotted in the form of a waterfall plot. The SNV with the highest AF (and at least above our technical threshold of 0.5%) in a patient at a given time-point (y-axis) across all fluid types (x-axis) are represented. Samples in which a CNA was detected are coloured red, whilst copy number neutral samples are coloured grey. The sensitivity of detection of CNAs using sWGS is considered to be ~5%^27^, and it is noteworthy that above this threshold (as indicated by SNV AF) all samples had detectable CNA. CNAs were also observed in samples in which the maximum SNV AF observed was <0.05%. It may be that these samples are primarily driven by CNAs and carry sub-clonal SNVs whose AF representation in plasma or urine are diminished as compared to tumours in which SNVs are clonal. Alternatively, these samples may have more extreme CNAs and are therefore more, easily detected. There was no clear difference in the relationship between CNA and SNV AF across the peripheral sample types tested.

Supplementary Figure 11: Longitudinal dynamics of patient specific SNVs and CNAs. A. Patient specific SNV kinetics across PLS, UCP and USN samples. Three plots are shown depicting longitudinal SNV kinetics across PLS, UCP and USN. Only the SNV with the highest AF at a given time-point is plotted, with line colours corresponding to individual patients. As demonstrated in Figure 3C, there are clear differences in the AF kinetics between peripheral sample types. Furthermore, there are differences in the kinetics between, and within, patients. For example, in patient 26, mutDNA levels at time-points 2 and 3 vary between UCP, where levels decrease, and USN, where levels increase. B. Changes in patient specific ‘genome-wide imbalance scores’ between first and last time-points, across peripheral sample types. Scores were calculated as described in the methods section but, briefly, involved linear modelling of autosomal 1Mb bin read counts in samples against those in a control sample, and subsequent summation of the 5% most extreme residual values (i.e. greatest difference vs. copy number neutral). Two predominant trends are observed; firstly, a series of patient samples that have high levels (>7) of CNA at their first time-point but much reduced levels at their final time-point. Of note, with the exception of patient 33, this series was enriched for patients that recurred early. The second trend observed involves ‘genome-wide imbalance scores’ that persist at low levels (<7) throughout NAC. Overall, the magnitude of CNA signals was higher in USN as compared to UCP, while levels were very low in PLS. Note - inset expands the low imbalance score range.

Supplementary Figure 12: SNV mutDNA kinetics for each peripheral sample type across the 6 patients who recurred early. Generally all 6 patients had high levels of mutDNA (most often) in their urinary samples. The format of these figures match the format described in Figure 5A. Notably, SNV kinetics of patient 9 were low in all sample types, including TUR indicating that the PIK3CA SNV that we track was not a component of the major clone. * there was no TUR sample for patient 15.

Supplementary Figure 13: SNV mutDNA kinetics for each peripheral sample type across the 6 patients who were free from early recurrence. Kinetics demonstrate generally low levels of mutDNA in all patients regardless of sample type. The format of these figures match the format described in Figure 5A.

Supplementary Figure 14: SNV mutDNA kinetics for each peripheral sample type of patient 11, who died shortly after surgery from surgical complication. The format of this figure matches the format described in Figure 5A.


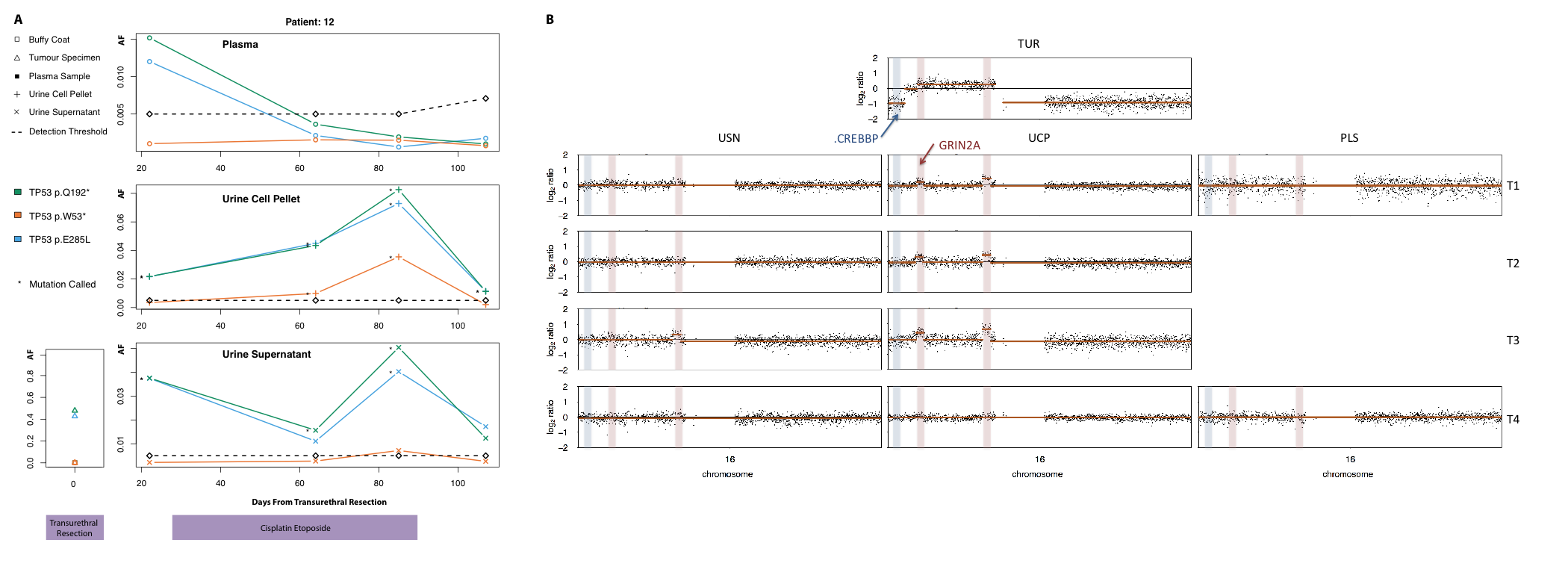


Supplementary Figure 15: MutDNA kinetics of patient 12 reveal tumour evolution on-therapy. A. Kinetics of SNVs in longitudinally obtained peripheral samples from patient 12. Changes in SNV AFs are plotted for PLS, UCP and USN from patient 12. SNVs in *TP53* (Q192* and E285L) are at their highest levels (as determined by AF) in PLS at time-point 1 but decrease below detection thresholds during NAC. Meanwhile, in UCP and USN, these mutations reach a peak AF at the third time-point (85 days after TUR). Coincident with this peak is the emergence of a third mutation of *TP53* (W53*). This mutation, which was not detected in the original TUR, reaches a modest AF of 3.6% in UCP and 0.7% in USN before receding at the final time-point (along with the other two *TP53* mutations). Despite its brief appearance, this third SNV indicates the emergence of a new clone under the selective pressure of NAC. B. Evidence of changing clonal dynamics during NAC through CNA analysis. Coincident with the emergence of *TP53* W53*, is the detection of a change in patient 12’s CNA profile. At TUR, the genome-wide CNA profile consists of multiple amplifications and losses involving both large chromosomal regions (sometimes whole chromosomes) and focal areas. This is exemplified by chromosome 16 (plotted), where 16p contains neighbouring regions showing focal loss (region shown in blue - including the *CREBBP* gene^24^), and large-scale amplification respectively. In peripheral samples, the TUR CNA profile (chromosome 16 and genome-wide) is largely absent. However, focal amplifications of two regions on 16p are observed, one of which contains *GRIN2A* (regions shown in red). The amplitude of these CNAs reached their peak at time-point 3 (in UCP) before receding at time-point 4. It is unclear if the focal amplification of GRIN2A is present in USN (low level CNAs) and PLS (time-points missing). Combined, the SNV and CNA data point to the emergence of a clone containing a *TP53* W53* SNV and a focal amplification of *GRIN2A*. It is unclear whether this clone has any influence on ultimate disease course in this patient who ultimately developed brain metastases after radical radiotherapy. Nonetheless, our data demonstrate the potential of mutDNA (in this patient, primarily derived from UCP) in tracking tumour evolution during therapy.

Supplementary Figure 16: Patient 15 CNA profiles of all three peripheral samples taken pre-NAC (T1), prior to Radical Cystectomy and lymph node dissection (T6) and from the radical cystectomy specimen. At T1 a CNA profile consisting of multiple amplifications and losses is obvious in the USN sample. Amongst these is a focal gain of *YAP1* on chromosome 11 which is also called in the matching UCP sample. At time-point 6, a completely different CNA profile is observed in USN (including focal loss of *CDKN2A* on chromosome 9). The UCP and PLS samples at this point are largely copy number neutral (though may show evidence of low-level *CDKN2A* loss). The cystectomy sample contains a similar CNA profile to that of USN T6, with focal *CDKN2A* loss and amplification chromosome 1q. The dynamics suggested by these profiles match those observed through SNV analysis at the same time-points. Combined, this data points to changing clonal dynamics, with the emergence of a new clone following initiation of NAC. Simultaneously, the clone that was dominant at T1 recedes, suggesting that it may be sensitive to NAC. Indeed, of all patients considered in this study, patient 15 has the highest SNV AF at the final time-point (Supplementary Figure 11) and examination of their clinical notes confirmed that they did not respond to therapy and later recurred. Therefore, mutDNA analysis of peripheral samples (here, in particular USN) can guide tracking of tumour evolution and disease course.

Supplementary Figure 17: mutDNA kinetics for patients with a broader spectrum of urothelial cancers. The format of these figures match the format described in Figure 5. A. For patient 1 (ureteric UCC), SNV kinetics differ between the urinary component and the plasma, with initially moderate levels in the former that decrease during NAC before re-appearing in the later time-points. PLS samples have generally low mutDNA kinetics throughout with low level mutDNA detected at 118 days following tumour biopsy B. For patient 30 (renal pelvis UCC), clonal dynamics differed across the 3 sample types, in samples collected over the period of 1 week. TP53 p.G279E mutDNA levels in PLS and USN levels declined whilst remaining stable in UCP samples. However, the clone containing the PIK3CA p.E542K mutation increased in the urinary components whilst decreasing in the PLS samples. C. mutDNA kinetics based on a genome wide imbalance score (GWIS) in patient 10 (metastatic UBC) demonstrate concordant kinetics in the urinary components, with an initial spike in levels soon after the initiation of NAC and a subsequent decrease during NAC. D. Grid depicting mutDNA detection across the 3 additional patients and time-points. The format of this grid matches that of the Figure 1C.

S1 Table: Demographics and treatment details of 17 MIBC patients with summary statistics. The table is in the same format as Table 1. Cystoscopy findings: 0 - no disease present, 1 - equivocal findings, 2 - small tumour (<3cm), 3 - large tumour (>3cm), ND - not done. Maximum AFs demonstrate the highest mutant AF of any mutation at the first time-point from any sample type. Definitive Treatments: RC - Radical Cystectomy, CR – Chemoradiotherapy, Rad – Radiotherapy, LND – Lymph Node Dissection. Response categories: CR- complete response, PR - partial response, SD - stable disease, PD - progressive disease. Other*: patient died from postoperative complications. +censured at the date of new tumour diagnosis.

S2 Table: Detection of mutDNA in pre-NAC samples did not correlate with response to NAC. Response categories: CR - Complete response, PR - Partial response, SD - Stable disease, PD - Progressive disease.

S3 Table: Patient demographics for additional patients are shown in a similar format to Table 1. Patient 1 had Urothelial Cell Carcinoma (UCC) of the distal ureter, patient 10 had metastatic UCC of the bladder and patient 30 had UCC of the renal pelvis. For patient 1 tumour DNA was extracted from biopsy tissue, for patient 10 DNA was extracted from TUR material prior to the diagnosis of metastatic disease, and for patient 30 DNA extraction was not possible due to the small amount of material obtained from the renal pelvis biopsy . All patients were treated with MVAC (methotrexate, vinblastine, doxorubicin and cisplatin). In addition, patient 1 had radical nephrouretectomy with lymph node dissection (NU + LND).

S4 Table: Grid depicting mutDNA AFs across all patients and time-points. The format of the grid matched that of Figure 1C. Where tested, data for CNAs were entered manually as 1 or 0 depending on whether or not they were called.

S5 Table: Grid depicting total genomic equivalent copies inputted per reaction across all patients and time-points. The format of the grid matched that of Figure 1C.
